# Hypusination, a Metabolic Posttranslational Modification of eIF5A in Plants during Development and Environmental Stress Responses

**DOI:** 10.3390/plants10071261

**Published:** 2021-06-22

**Authors:** Péter Pálfi, László Bakacsy, Henrietta Kovács, Ágnes Szepesi

**Affiliations:** Department of Plant Biology, Institute of Biology, Faculty of Science and Informatics, University of Szeged, Közép Fasor 52., H-6726 Szeged, Hungary; palfipeter98@gmail.com (P.P.); bakacsy@gmail.com (L.B.); henrietta.kovacs96@gmail.com (H.K.)

**Keywords:** hypusination, polyamines, eIF5A, deoxyhypusine synthase, deoxyhypusine hydroxylase, metabolic PTM

## Abstract

Hypusination is a unique posttranslational modification of eIF5A, a eukaryotic translation factor. Hypusine is a rare amino acid synthesized in this process and is mediated by two enzymes, deoxyhypusine synthase (DHS) and deoxyhypusine hydroxylase (DOHH). Despite the essential participation of this conserved eIF5A protein in plant development and stress responses, our knowledge of its proper function is limited. In this review, we demonstrate the main findings regarding how eIF5A and hypusination could contribute to plant-specific responses in growth and stress-related processes. Our aim is to briefly discuss the plant-specific details of hypusination and decipher those signal pathways which can be effectively modified by this process. The diverse functions of eIF5A isoforms are also discussed in this review.

## 1. Introduction

Protein synthesis and protein modifications are important processes that help plants survive in harsh conditions. Different types of posttranslational modifications (PTMs) of proteins exist in plants. However, hypusination is unique because it is limited to only one protein, the eukaryotic translation factor 5A known as eIF5A (reviewed in [1]). This modification is catalysed by two sequential enzymatic steps leading to the activation of eIF5A by the conversion of one conserved lysine to the unusual amino acid hypusine [2]. The first step is dependent on a triamine polyamine, called spermidine (Spd), which provides the 4-aminobutyl moiety group destined to the lysine of eIF5A, forming an intermediate deoxyhypusine (Dh-Hyp). Each of the enzymes in the hypusination is coded by only one gene, respectively. However, eIF5A is encoded by a small multi-gene family. The Spd-mediated hypusination is essential in eukaryotes, demonstrating one of the main functions of polyamines (PAs) in plant responses from development to stress [3]. eIF5A plays an important role in regulating translation under stress conditions in eukaryotic cells and may be critical in adapting plants to prevailing environmental conditions. The disruption of hypusination leads to growth arrest in proliferating eukaryotic cells and is fatal for the developing mammalian embryo. Plant eIF5A proteins are also highly conserved and are involved in multiple biological processes, including protein synthesis regulation, translation elongation, mRNA turnover and decay, cell proliferation, leaf and root growth, seed yield, leaf, flower and fruit senescence, and programmed cell death [4,5,6]. eIF5A is necessary for mRNA translation and translocation from the nucleus to the cytoplasm [7]. eIF5A promotes the translation of polyproline [8] and other non-polyproline stalling motifs ([9,10] which are poor substrates for protein synthesis, causing ribosome stalling. The identification of mRNA targets depending on eIF5A in plants was started by [11]. Mandal et al. (2014) [12] compared the numbers of PPP and PPG motifs in proteins using phylogenetic analysis and found that these motifs increased via the developmental level of the organisms, indicating that the complexity of an organism contributes to more functions that need these motifs for regulation. In Arabidopsis, more than 3500 proteins contain at least three consecutive proline (Pro) in their structure. These proteins gained new specialized functions as products of late evolutionary events, contributing to the better adaptation of plants to changing conditions [2,12,13]. The newest results show that Spd, through its involvement in hypusination, can act as a molecular sentinel to prevent translation anomalies that may lead to activation of the nonsense-mediated decay (NMD) machinery and subsequent mRNA degradation [14].

In this review, we focus on representing the hypusination process and plant-specific isoforms of eIF5A associated with growth, development, and stress responses. The recent findings on hypusination and the diverse role of eIF5A isoforms in plants and their animal counterparts will also be discussed. 

## 2. Polyamine-Dependent Hypusination of eIF5A in Plants 

**Figure 1:**
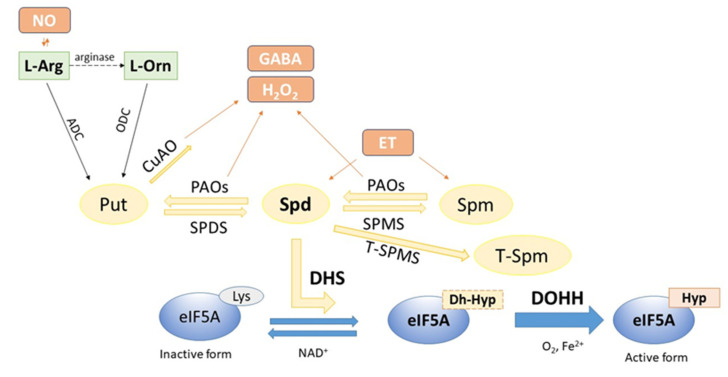
A schematic overview of the Spd-mediated hypusination process of eIF5A in plants and related pathways. Spd is essential for the DHS reaction to produce deoxyhypusine (Dh-Hyp) on inactive forms of eIF5A via cofactor NAD^+^. Spd is derived from both the PA biosynthesis catalysed by Spd-synthase (SPDS) and catabolism by polyamine oxidases (PAOs). It is worth mentioning that the metabolism of Spd is strongly connected to the biosynthesis of important signal molecules such as nitric oxide (NO), hydrogen peroxide (H_2_O_2_), gamma-aminobutyric acid (GABA), and ethylene (ET) providing routes for the complex influence of Spd levels in plants. The second step of hypusination is catalysed by DOHH, which needs oxygen and diiron for its function, in order to produce the hypusine of mature eIF5A. Abbreviations: Put—putrescine; Spm—spermine; L-Arg—L-arginine; L-Orn—L-ornithine; ADC—arginine decarboxylase; ODC—ornithine decarboxylase; CuAO—copper amine oxidase; SPMS—spermine synthase; T-SPMS—thermospermine synthase.

Polyamines (PAs) are essential polycations with crucial roles in the growth, development, and stress responses of all living organisms [3]. The main PAs in plants are diamine putrescine (Put), and the higher PAs spermidine (Spd) and tetraamine spermine (Spm). Polyamine metabolism is a fundamental process in all living organisms which is connecting with other important pathways regulating growth and development and also stress responses [15,16,17]. The triamine Spd is essential in cell growth and development, as mutant plants with depleted Spd suffer embryo death [18]. Spd is the substrate for DHS, the first enzyme involved in hypusination (Figure 1).

As a precursor for hypusine, it is also speculated that the correct Spd/Spm ratio could be critical in efficient hypusination processes [19]. The level of Spd is strongly influenced by its biosynthesis from L-arginine by arginine decarboxylase (ADC) or L-ornithine by ornithine decarboxylase (ODC), depending on the plant species. eIF5A promotes the alleviation of ribosome stalling, contributing to the translation of mRNAs, e.g., in humans, of the oncoprotein MYC. This contributes to the proliferation of colorectal cancer cells [20]. MYC could regulate ODC enzyme translation, causing an increase in the PA level. Spd thus can enhance the hypusination of eIF5A, creating an amplification feedback loop in animal organisms [20]. As ODC cannot be found in several plants, it would be interesting to compare plants with different PA biosynthesis mechanisms regarding how the presence of ODC could affect the hypusination of eIF5A and modify the amplification feedback mechanisms. Recently, it was discovered that the suppression of ribosomal stalling by eIF5A is necessary for the fidelity of the start codon preference, which suggests that eIF5A activity could affect PA availability via translational reprogramming [21]. However, this needs further investigation in plants.

The complex involvement of PA-dependent hypusination of eIF5A in ribosomal quality checks is described by Poidevin et al. (2019) [14]. Defects in the Spd-dependent hypusination of eIF5A could result in ribosome stalling and the activation of no-go decay (NGD) mechanisms, or could induce non-stop ribosomal activities [14] via inactive eIF5A in plants. New evidence revealed that Spd and thermospermine (T-Spm), the latter of which is essential for vascular development, could act as molecular sentinels during translational processes in order to avoid translational imbalances which could activate the nonsense-mediated decay (NMD) system and related mRNA degradations [14]. Currently, there is no evidence of the involvement of other PAs in this process in plants [14]. 

PA catabolism is also critical for regulating the Spd level used for eIF5A hypusination [22]. Many studies strongly suggest that Spd or oxidation products of PAs or conjugation can play a role in cell growth [23] related to other signal pathways such as ethylene (ET) or gamma-aminobutyric acid (GABA).

In plants, the Spd level is also connected with Pro and nitric oxide (NO) biosynthesis [24]. NO is also involved in other posttranslational modifications of proteins, namely S-nitrosylation. It is important to note the occurrence of PA-triggered S-nitrosylation of key protein interactions with ABA and redox signalling [3]. Additionally, L-arginine is an important source of PA biosynthesis, as well as of NO biosynthesis in several plants [25].

ABA-dependent regulation of eIF5A hypusination in *Arabidopsis thaliana* was also uncovered by Belda-Palazón et al. (2014) [11]. ABA is a key hormone regulating and controlling the onset of leaf senescence, coordinating the Ying–Yang of plant life [26]. eIF5A in *Picrorhiza*, which is adapted for shading environment, is up-regulated during leaf senescence and in response to ABA [27], indicating that eIF5A has a role in leaf senescence mediated by ABA. This study also demonstrates that eIF5A could be an important candidate for the development of genetic engineering-based strategies for delaying leaf senescence.

Spd is essential in cell growth and development, but the Spd-dependent growth is not only associated with its role in eIF5A hypusination [3] as the eIF5A pool starts to decrease when cell division is inhibited by PA depletion.

There are some open questions regarding hypusination in plants. For example, Spd could be a rate-limiting step in this process. We do not know exactly which forms of Spd could be used for hypusination—free, bound, or conjugated. It is just speculation that the Spd concentration for hypusination could be decreased to a greater extent than the total PAs. During investigations with yeasts, it was demonstrated that in Spd-deficient conditions, half of the retained Spd can be used for hypusine formation, suggesting that there is a constant ratio that is necessary for maintaining the hypusine pool. 

## 3. Two Steps of Hypusination

During hypusination, two enzymatic reactions bring eIF5A to its active form: First, in humans, deoxyhypusine synthase (DHS, EC.2.5.1.46) catalyses the transfer of the 4-aminobutyl moiety of Spd to the ε-amino group of conserved Lys50 to form an intermediate residue, deoxyhypusine (Dhp50). Subsequently, deoxyhypusine hydroxylase (DOHH, EC1.14.99.29) mediates the formation of hypusine (Hyp50) via the addition of a hydroxyl group to the deoxyhypusine residue. eIF5A, DHS, and DOHH are all essential for the proliferation of higher eukaryotic cells, and the eIF5A protein is strictly conserved throughout eukaryotic evolution [28,29].

### 3.1. First Step: Synthesis of Deoxyhypusine by Deoxyhypusine Synthase (DHS)

DHS is the first enzyme involved in the hypusination process of eIF5A. Some results show the significance of this rate-limiting step in hypusination. DHS is strongly conserved in eukaryotes. In animal organisms, it catalyses the transfer of the aminobutyl moiety of Spd to the ε-amino group of highly conserved lysine-50 in the eIF5A precursor protein. The deoxyhypusine-eIF5A is further converted to active hypusine-eIF5A, which is essential for the transport of newly transcribed mRNAs from the nucleus to the cytoplasm [30]. The Arabidopsis plant’s mutant in the DHS gene possesses a lethal female gametophyte and cannot be a homozygous diploid [31]. Evidence shows that the expression of DHS could spatially and temporally change via the developmental stages. In Petunia, suppression of DHS caused abnormal chloroplast development in the leaves and delayed leaf senescence [32]. However, in Arabidopsis, the leaf-specific suppression of DHS resulted in growth enhancement without any negative pleiotropic effects [33] despite an earlier study that demonstrated the pleiotropic effects of suppressed DHS expression in the same plants [4]. Suppressed DHS caused increased root biomass, bigger leaves, and enhanced seed yield in Arabidopsis during drought stress [4]. Sectored chlorotic leaves were also detected in Petunia plants, suggesting that PhDHS is involved in their chloroplast development [32], indicating a functional difference between different plant species. DHS silencing caused extended leaf longevity and increased thickness of the leaves, with reducing sugar and starch contents indicating the compensation of energy loss. The silencing of DHS was accompanied by increased levels of proteins involved in ribosome complex composition, porphyrin and chlorophyll metabolism, and stilbenoid, diarylheptanoid, and gingerol biosynthesis [32]. In canola (*Brassica napus* cv. Westar), DHS suppression resulted in larger leaves, a greater abundance of siliques, and higher seed yield in transgenic canola lines and also exhibited enhanced tolerance to chronic sublethal stress. It is suggested that the steady-state concentration of different isoforms of eIF5A is important, as DHS downregulation caused enhanced growth, reflecting that growth-related isoform levels were higher than PCD-related isoforms. Stronger suppression of the DHS could induce the activation of cell-death-related isoforms. As canola is an important oil crop, cultivars with reduced DHS activity could be important to increase the oil production of plants without any significant reduction in the quality of oil [34]. 

Tomato DHS and eIF5A were upregulated during drought and chilling stress, coinciding with flower and fruit senescence [35]. It is suggested that plant DHS and eIF5A promote the translation of those mRNAs which are needed for inducing PCD; however, they are not involved in the regulation of nutrient mobilization [35]. The aminobutyl moiety of Spd is also transferred to Put via the action of homospermidine synthase (HSS). Several studies suggest the general occurrence of homospermidine in plants and its supposed origin as a by-product of DHS [36]. A high degree of sequence identity between DHS and HSS genes suggests that HSS evolved via gene duplication from DHS [37], which may have occurred at least four times in separate angiosperm lineages [38]. The resulting homospermidine is an essential precursor in the biosynthesis of pyrrolizidine alkaloids that serve as defence compounds against insect herbivores and occur in a number of families, including the Asteraceae, Boraginaceae, and Orchidaceae families [36]. The DHS-catalysed reaction is specific for Spd binding as the chain length is sufficient to bind to the active site of the enzyme which no other PAs can bind to. When Spd and eIF5A bind to the DHS, its N-terminal part goes through a conformational change which is necessary for its activity [39]. Thompson et al. (2003) [40] suggested that the α-helix in the N-terminal of the DHS could inhibit the binding of eIF5A when this protein is not needed. Some promising novel DHS inhibitor compounds could be effective for modifying the hypusination of plants to enhance their abiotic stress tolerance. Conformational dynamics are also important in the regulation of DHS and eIF5A hypusination [41]. Results indicate that the cofactor and NAD^+^ concentration can regulate proper NAD^+^ binding to DHS. However, to date, the exact concentration which is needed for optimal hypusination has not been investigated in plants. 

### 3.2. Second Step: Synthesis of Hypusine by Deoxyhypusine Hydroxylase (DOHH)

DOHH (EC EC1.14.99.29) is a non-heme diiron oxygenase that has only one substrate, the deoxyhypusine of eIF5A. This enzyme mediates the formation of hypusine via the addition of a hydroxyl group to the deoxyhypusine residue in an irreversible manner, which means that this reaction positively and irreversibly affects hypusination. This process requires oxygen and iron for the efficient functioning of the enzyme. The iron dependence of this enzyme was evidenced by inhibitors which initiated a chelation process with the ferrous iron of DOHH in *Plasmodium falciparum* [42]. In animal counterparts, some iron-dependent chaperons were identified which are able to directly transfer the iron to the enzyme to be incorporated in a posttranslational manner [43]. To date, the investigation of plant-specific iron chaperons remains to be undertaken. 

## 4. Functions of eIF5A in Plants

Hypusination is required for RNA binding and protein interaction. All eIF5A members contain a nucleotide-binding motif [44] and RNA binding which is hypusine dependent [45]. In the case of Spd depletion, growth arrest and decreased levels of hypusine occurred in yeasts. Inhibitors of the hypusination of eIF5A caused the elimination of some specific mRNAs from polysomes, indicating the selective nature of eIF5A in translation. Nuclear-cytoplasmic shuttling occurred via exportin-4 interaction, which is a nuclear export receptor in animals [46]. eIF5A specifically binds to the trimeric eIF5A-Exp4-Ran –GTP export complex which produces its efficient nuclear export.

eIF5A can promote the translation of mRNAs by transferring them from the nucleus [47]. eIF5A can also act as a transcription factor in the nucleus as it can bind to a promoter and regulate some gene transcriptions [48]. As eIF5A isoforms can be found in the nucleus as well as in the mitochondria, their function should be investigated to decipher the precise role of this protein. 

If the eIF5A level was decreased in yeast intracellularly, specific mRNAs accumulated at high levels in the nucleus [35] as their transport from the nucleus was inhibited, suggesting that mRNA translocation is one of the important roles of eIF5A proteins. Other types of PTM such as phosphorylation also can affect the efficacy of eIF5A [49]. The N-terminal sequence of the plant eIF5A contains a consensus motif for casein kinase 2 (CK2), demonstrating that phosphorylation can occur; however, the precise role of this modification is not well understood in plants [13]. eIF5A could enter the nucleus via passive diffusion. However, phosphorylation can inhibit exportation from the nucleus. The phosphorylation of the nucleocytoplasmic shuttling of eIF5A could be a unique physiological mechanism that can regulate the specific nuclear export of mRNAs bounded by eIF5A [49]. Moreover, the phosphorylated or non-phosphorylated eIF5A could play different roles in plant development and stress responses [6]. 

Pumpkin eIF5A isoforms interact with components of the translational machinery in the cucurbit sieve tube system [50]. All three isoforms were detected in pumpkin phloem sap, indicating the independent entrance of the isoform into the phloem without hypusination. Both modified and non-modified eIF5A forms were studied in the phloem sap, so DHS and DOHH could also be found, demonstrating that PTM could occur inside the sieve elements [50]. 

eIF5A is able to interact with ribosomal proteins and phloem proteins during protein synthesis [50]. Spd, which is essential for this PTM, is also found in sieve elements. CmeIF5A could bind nonspecifically to any RNA which can be found in the phloem transport. It is important to mention that CmeIF5A-2 and CmeIF5A-4 show functional redundancy via similar protein binding activity; however, in Arabidopsis, this type of redundancy is not found between isoforms [28]. The study of more species-specific isoform functions could be helpful for discovering species-specific hypusination in plants.

Plant eIF5A genes are also involved in abiotic stress responses. However, little is known of the upstream regulators, or of the regulatory network and its role in stress tolerance. In addition, if eIF5A does in fact confer stress tolerance in plants, the physiological changes mediated by eIF5A deserve further study. Studying AveIF5A from *Apocynum venetum*, a halophyte shrub, showed that its expression profile was different for diverse stress conditions, such as cold, salt or drought stress [51]. These results indicated that eIF5A could play an important role in some abiotic stress conditions, and the timing of stress could imply the success of eIF5A-promoted tolerance processes. 

Several defects in hypusination could be restored by exogenous Spd, as studied in mice by Schroeder et al. (2021) [52]. The newest results indicate that impaired eIF5A hypusination can cause serious Mendelian disorder in humans, which could be partly restored by Spd supplementation [53]. It is important to note that during Spd supplementation, neither the eIF5A expression nor hypusination increased, suggesting a potential mechanism and additional role of this PA. Spd has more functions that are needed for optimal cell growth and development. It is an essential substrate for the hypusination of the eIF5A protein and is also involved in translational fidelity in animals [54]. Exogenous Spd supply could be useful for restoring eIF5A efficiency as it can contribute to PA increase, which could also increase the general translational efficacy. This could be helpful for studying the hypusination process involved in stress tolerance enhancement while Spd is supplied in plants. 

eIF5A was identified via deep sequencing as a key element in soybean mosaic virus (SMV) resistance gene 1, the Rsv1-mediated lethal systemic hypersensitive response to soybean mosaic virus infection in soybeans [55]. Infection with the virus-induced transcriptional alterations accompanied by different morphological changes in the plants. In soybeans, there are seven loci coding eIF5A homologues, as a genome duplication occurred twice in this plant [56]. Interestingly, only one homologue was induced after infection. However, it is suggested that other homologues were also silenced. In silenced plants, both pathogenesis-related gene 1, PR1 gene expression, and ROS production decreased, indicating that eIF5A can act upstream of ROS and PR1 [55].

Transcript encoding translation factor eIF-5A is stored in the unfertilized egg cells of maize [57], suggesting that the function of this protein is to recognize the target transcripts which are stored in unfertilized egg cells and selectively translate after fertilization, as in mature egg cells this protein is not detected. Hopkins et al. (2008) [58] reported that eIF5A plays a vital role in signal transduction pathways involved in pathogen-induced cell death and in the development of plant disease symptoms. mRNA levels of DHS and eIF5A correlate during growth and development. The upregulation of DHS could influence flower senescence, tomato fruit softening, and premature leaf senescence, which are induced by environmental stresses [59]. In tomato plants, there are four members of SleIF5As. Suppressed DHS causes a delay in postharvest development, but this has no effect on fruit ripening in tomatoes [59]. Given the fact that only one SlDHS gene can take part in the posttranslational activation of all SleIF5A isoforms and transgenic plants with suppressed DHS did not reflect the knockout of only one eIF5A isoform, it can be concluded that there may be functional redundancy between SleIF5A isoforms. However, this idea needs to be experimentally investigated under different stress conditions. Additionally, to date, there is no evidence regarding the level of functional redundancy in senescence processes in plants. 

Puleston et al. (2019) [60] showed that the biosynthesis of PAs in animal organisms could modulate mitochondrial metabolism, especially oxidative phosphorylation, through the eIF5A hypusination. They found that some mitochondrial enzymes are dependent on eIF5A hypusination in their expression. There is no evidence in plants for this type of regulation in mitochondria, which could clarify another regulating aspect of PAs and hypusinated eIF5A. 

## 5. Diverse Roles of eIF5A Isoforms in Plants

The plant eIF5A isoforms have different roles in cell division and cell death [7] or stress tolerance. eIF5A was shown to act as a local or long-distance signal in pumpkins, suggesting that its isoforms can act in different developmental stages but can also show similar functions in sieve tube systems [50]. Some proteins were found to act as interacting proteins with *Cucurbita maxima* CmeIF5A, CmPP16-1, CmPP16-2, CmRBP50. 

This section will describe the diverse role of eIF5A isoforms in plants which have evolved along with the ancient function of hypusination.

### 5.1. eIF5A-1

Evidence shows that modulating eIF5A expression can alter xylem abundance in *Arabidopsis thaliana* [29]. The AteIF5A-1, which is located in the cytoplasm and extracellular region, is expressed in developing xylem tissues, promoting xylem development. It is suggested that AteIF5A-1 is able to influence auxin biosynthesis and transport, but this needs to be investigated. The non-hypusinated form of eIF5A is supposed to be involved in xylogenesis. Many xylem-related genes have been identified [61]. However, evidence suggests that non-hypusinated AteIF5A-1 can translocate those mRNAs which could encode for one or more genes responsible for xylem development [29].

One of the most studied eIF5A genes is TaeIF5A from *Tamarix androssowii* [62]. TaeIF5A1 is expressed in all tissues and some abiotic stress conditions enhance its levels. It was found that the expression of TaeIF5A1 is regulated by TaWRKY and TaRAV as both can bind to W-box. In the promoter region of TaeIF5A, a W-box-binding motif can be found, suggesting that it can be regulated by W-box binding transcription factors [62]. Both TaWRKY and TaRAV could activate the expression of TaeIF5A by binding to its W-box motif. All three genes show similar expression patterns and could be inhibited by ABA treatment induced by osmotic stress, strongly suggesting that these three genes are components of a regulatory pathway in plants. Additionally, TaeIF5A could enhance the level of SOD and POD enzyme activities and protein synthesis, providing evidence that ROS scavenging is an important function of this protein. Chlorophyll loss was also decreased during salt stress, enhancing the tolerance of the plants [62].

Three proteins could interact with TaeIF5A: a protein with an unknown function, XTH, and Arf GAP [63]. XTH is responsible for the cleavage of xyloglucan chains which are involved in the rearrangement of the cell wall [64]. Additionally, Valentini et al. (2002) [65] showed that eIF5A is involved in the cell wall integrity and stress response component (WSC/PKC1) signaling pathway that controls cell wall integrity or related processes and plays a role in cell wall formation. These results provide evidence that eIF5A is involved in cell wall biosynthesis. Another role of eIF5A is its involvement in intracellular trafficking via interaction with Arf GAP proteins, which are involved in the GTP-binding of the ARF/SAR family, and actin cytoskeleton reorganization [65,66,67]. This needs to be understood in plants. It is suggested that TaeIF5A1 has a different role than the other type of eIF5A [63]. 

The overexpression RceIF5A, which encodes a eukaryotic translation factor 5A in *Rosa chinensis*, could enhance the thermotolerance, oxidative, and osmotic stress resistance of *Arabidopsis thaliana* [6]. Additionally, SOD enzyme activity was increased, suggesting a protective role of eIF5A in stress defence. As Pro levels also increased via the elevation of biosynthesis Δ1-pyrroline 5-carboxylate synthase (P5CS) activities, it can be concluded that eIF5A can induce enhanced adaptability to abiotic stress conditions. The differential expression of genes encoding the elF-5A in tobacco was studied by Chamot et al. (1992) [68]. It was suggested that NeIF5A-2 could be a housekeeping protein that plays a role in general translation initiation; however, the other isoform, NeIF5A-1, could regulate the light-dependent translation of several transcripts. 

Cultivars with different salt tolerance levels were investigated [69]. In the case of *Hordeum vulgare*, it was found that eIF5A-1 isoforms are more abundant in *H. vulgare*, inducing apoptosis, while the salt-tolerant *H. maritimum* contained more eIF5A-2 isoforms responsible for cell division [70]. Wang et al. (2016) [51] conducted a study on *Apocynum venetum* eIF5A1, which is a salt-tolerant halophyte herb plant. These studies suggest a cultivar- and a species-dependent mechanism of hypusination and eIF5A isoforms in plants. However, more studies are needed to find functional differences of eIF5A isoforms and hypusination between cultivars differing in salt tolerance. These findings could be useful for biotechnological approaches to enhance the salt tolerance of crop plants (Table 1). 

### 5.2. eIF5A-2 or FBR12

Functional characterization of Arabidopsis eIF5A-2, which is located in the cytoplasm, cytosol and nucleus, suggests a crucial role in plant growth and development by regulating cell division, cell growth, and cell death [28]. eIF5A is involved in the induction of pathogen-induced cell death and the development of disease symptoms in Arabidopsis [58].

*AteIF5A-2* (*FBR12*) is involved in xylem development and is accumulated in root vascular tissues. FBR12 can interact genetically with histidine kinases, CRE/WOL, and the AHP2,3,5, CRE family receptors, suggesting an important role in cytokinin signalling. The AteIF5A-2 regulates root protoxylem development by modulating cytokinin signalling [71]. FBR12 could specifically regulate the repression of AHP6, which is a negative regulator of cytokinin signalling. Notably, fbr12 mutants show normal auxin signalling in protoxylem development. FBR12 could bind a complex with CRE1 and AHP proteins and can also regulate phosphorelay activities. The positive effect of FBR12 could come from two things: the enhancement of phosphorelay stabilizing the CRE1-AHP1 complex, and by antagonizing the inhibiting effect of AHP6 on the phosphorelay, indicating that FBR12 could be a bifunctional molecule in plants [71]. 

In *Arabidopsis thaliana*, the downregulation of *AteIF5A-2* (*FBR12*) caused increased cadmium (Cd) sensitivity in plants [72]. The fbr12 mutant accumulated Cd and ROS burst was induced, demonstrating that AteIF5A regulates the oxidative stress response of this plant under heavy metal stress. Additionally, this alteration affected some genes related to Cd transporter gene expression, long-distance transport, and detoxification; however, the precise mechanism of how eIF5A regulates the genes of phytochelatin synthesis is not well understood. Moreover, the precise mechanism of how Cd sensitivity is influenced by eIF5A during Cd accumulation is unknown. Studying this connection could elucidate a possible defence strategy of plants against Cd. 

The expression of genes encoding the rice translation factor, eIF5A, is involved in developmental and environmental responses [73]. Both OseIF5A genes might be regulated by plant development and environmental stresses, as Chou et al. (2004) [73] reported that salt and heavy metal stresses induce the expression of rice eIF5A genes, OseIF5A-1 and OseIF5A-2, suggesting their involvement in stress tolerance (Table 1).

### 5.3. eIF5A-3

In Arabidopsis, the third isoform is eIF5A3, which is located in cytoplasm, cytosol and mitochondria. Gain-of-function mutants of Arabidopsis eIF5A-3 induced a seed yield enhancement influencing the growth and response to osmotic and nutrient stresses [50]. Additionally, AteIF5A-3 plays a role in auxin hormone translocation; however, it was not found in meristematic tissues, showing tissue-specific localization. It can be concluded that AteIF5A-3 has a more dominant role in nutrient and hormone translocation than in cell division [5]. In yeasts, Barba-Aliaga et al. (2020) [74] described the different regulation activities of eIF5A isoforms during iron starvation, as eIF5A-1 was downregulated while eIF5A-2 was upregulated. Under Fe-deficient conditions, a generally decreased abundance of ribosomal proteins was investigated. However, it was evidenced that eIF5A3 was responsive to Fe deficiency in order to help reprogram the synthesis of proteins involved in growth [75]. 

As we can see from the results discussed above (Table 1), different isoforms of eIF5A could induce stress tolerance or programmed cell death depending on the conditions. It is tempting to speculate that these isoforms could act together in some conditions, and this could be visible in some overlapping responses [6]. It would be helpful to determine the steady-state concentration of different isoforms of cultivars with different stress sensitivity in order to determine the efficacy of hypusination under different levels of stress. Suppression of hypusination could downregulate all three isoforms in plants. To date, there is no evidence for calculating which isoforms could be active at the same time in response. Maybe, new methods and metabolomics studies will help to decipher the interplay of different isoforms in variable conditions. 

## 6. Potential Biotechnological Application of eIF5A-Related Processes of Plants

New evidence suggests that hypusination, as an Spd-dependent metabolic pathway, could play an important role in the translation of inducible antimicrobial effectors of macrophages by bacteria in animals [76]. In plants, pathogenic bacteria could influence also the hypusination process but to date, these processes are not investigated. These results could achieve new roles of hypusination in plants uncovering some important new roles of eIF5A mediated hypusination.

Given the fact that the hypusination and PA-eIF5A nexus are conserved in all eukaryote organisms, these processes could be excellent targets of manipulation of plant–pathogen interactions in agriculture. It was evidenced that imbalances in eIF5A hypusination in *Fusarium graminearum*, a fungal pathogen of cereals causing decreased food safety by toxin production and yield loss, could be effective for modifying the pathogenicity and toxicity of this fungus [77]. Increased hypusinated eIF5A in DOHH overexpressed mutant fungi resulted in reduced toxin production and virulence, demonstrating the potential application to minimize the yield loss and toxin concentration of cereal crops. 

There are some medicinal plants that contain toxic pyrrolizidine alkaloids, limiting their pharmacological application. The latest results indicate that the Crispr/Cas9 system was effective in HSS knockout, so the plants did not contain any toxic alkaloids, contributing to their safety [78]. In the future, this approach could provide a new way to modify toxin-containing medicinal plants for safe use in medicine. 

New assays for detecting NADH at sub-micromolar levels provide a non-radioactive method to measure DHS activity in biological samples, contributing to the search for new inhibitor compounds for hypusination [79]. 

## 7. Concluding Remarks and Perspectives

Hypusination represents a unique metabolite-dependent posttranslational modification of eIF5A in eukaryote organisms. This conserved process contributes to the formation of active eIF5A, which could participate in diverse plant responses from growth and development to stress-induced tolerance. The involvement of this protein as a switch for inducing stronger adaptation to diverse conditions triggering cell division or cell death, as well as its participation in stress responses, is well established. However, the precise mechanism by which eIF5A hypusination is influenced by its essential substrate, Spd, requires further study. The ratio of hypusinated and non-hypusinated eIF5A isoforms, and also those Spd forms which are most suitable for hypusination in plants, has yet to be determined. This is because eIF5A can be in free form, conjugated, or bounded. Identifying mRNA targets depending on eIF5A could help us elucidate new aspects of the significance of eIF5A hypusination in plants. An amplification feedback loop between eIF5A and PA homeostasis is suggested; however, it needs to be confirmed in plants. The picture is more complex considering that PA homeostasis is strongly affected by the biosynthesis and degradation of other signalling molecules and phytohormones, providing another level of activation of eIF5A. Different components of this modulation need to be identified in future studies involving different omic techniques and metabolomics approaches. Additionally, the several posttranslational modifications which could fine-tune the hypusination and other effects of eIF5A are still matters of further inquiry. Studying different plant species, hypusination, and isoforms of eIF5A can help to indicate species-specific responses which could be useful for biotechnological approaches to enhancing crop yield and crop protection, food safety, and stress tolerance of plants, and these results could be used in agriculture, food, or the pharmaceutical industry. 

## Figures and Tables

**Table 1:** The diverse roles of eIF5A isoforms in *Arabidopsis thaliana*.

Protein Isoform	Synonyms	Protein Accession No.	Biological Process	Ref.
eIF5A-1	Atelf5A-1 eif-5A eif5A, elf5A-1 eukaryotic elongation factor 5A, eukaryotic elongation factor 5A-1	AT1G13950	shuttle protein translocating mRNA from the nucleus to cytoplasmic ribosomes Xylem formation	[29,33]
eIF5A-2	Atelf5A-2, elf5A-2 eukaryotic elongation factor 5A-2, FBR12 fumonisin B1-resistant12	AT1G26630	Involved in programmed cell death triggered as a response to pseudomonas syringae infection. The mRNA is cell-to-cell mobile	[58,71]
eIF5A-3	Atelf5A-3, elf5A-3, eukaryotic elongation factor 5A-3	AT1G69410	Growth during osmotic and nutrient stress	[5]
